# Usefulness of Bnet, a Simple Linear Metric in Discerning Torsades De Pointes Risks in 28 CiPA Drugs

**DOI:** 10.3389/fphar.2019.01419

**Published:** 2019-11-26

**Authors:** Sungpil Han, Seunghoon Han, Ki-Suk Kim, Hyang-Ae Lee, Dong-Seok Yim

**Affiliations:** ^1^Department of Clinical Pharmacology and Therapeutics, Seoul St. Mary’s Hospital, Seoul, South Korea; ^2^PIPET (Pharmacometrics Institute for Practical Education and Training), College of Medicine, The Catholic University of Korea, Seoul, South Korea; ^3^R&D Center for Advanced Pharmaceuticals & Evaluation, Korea Institute of Toxicology, Korea Research Institute of Chemical Technology, Daejeon, South Korea

**Keywords:** proarrhythmic risk, biomarker, torsade metric score, Bnet, ion channel, CiPA, ICH

## Abstract

The Comprehensive *in vitro* Proarrhythmia Assay (CiPA) project suggested the torsade metric score (TMS) which requires substantial computing resources as a useful biomarker to predict proarrhythmic risk from human ether-à-go-go–related gene (hERG) and a few other ion channel block data. The TMS was useful to predict low TdP risks of drugs blocking Na^+^ (ranolazine) and Ca^2+^ (verapamil) channels as well as the hERG channel. However, Mistry asserted that the simple linear metric, Bnet reflecting net blockade of a few influential ion channels has similar predictive power. Here we compared the predictability of Bnet and TMS for the 12 training and 16 validation CiPA drugs which were pre-classified into three categories according to the known TdP risks (low, intermediate, and high risk) by CiPA. Bnet at 5×C_max_ (Bnet_5×Cmax_) was calculated using the ion-channel IC50 and Hill coefficients of CiPA drugs collected from previous reports by the CiPA team and others. The receiver operating characteristic curve area under curve (ROC AUC) values for TMS and Bnet_5×Cmax_ as performance metrics in discerning low versus intermediate/high risk categories for the 28 CiPA drugs were similar. However, Bnet_5×Cmax_ was much inferior to TMS at discerning between intermediate- and high-risk drugs. Dynamic Bnet, which used *in silico* hERG dynamic parameters unlike conventional Bnet, improved the misspecification. Thus, we propose that Bnet_5×Cmax_ is used for quick screening of TdP risks of drug candidates and if the “intermediate/high” risk is predicted by Bnet_5×Cmax_, *in silico* approaches, such as dynamic Bnet or TMS, may be further considered.

## Introduction

The International Council on Harmonization (ICH) established the guidelines, S7B for non-clinical evaluation and E14 for clinical evaluation of the proarrhythmic risk of drugs. As recommended by the guidelines, the conventional practice to evaluate the Torsades de Pointes (TdP) risks has been focused on the QTc interval from blockade of human ether-à-go-go–related gene (hERG) channel (Shah, 2005) that is associated with rapidly activating delayed rectifier potassium current I_Kr_. (Sager et al., 2014) Although ICH S7B and E14 regulatory guidelines have been successful in screening TdP risks of new drugs, there are several low TdP risk drugs with the prolonged action potential duration (APD) and QTc interval. Thus, the current practice according to ICH guidelines is sensitive but not specific enough to evaluate proarrhythmic (TdP) risks.

One of the major objectives of the Comprehensive *in vitro* Proarrhythmia Assay (CiPA) initiative was to improve the current ICH guidelines to avoid the misclassification of TdP risks by evaluating mechanistically based *in vitro* assays and *in silico* reconstruction of the cardiac action potential. The CiPA ion channel working group and *in silico* modeling group suggested qNet and the torsade metric score (TMS) as conclusive markers *via* the CiPAORdv1.0, the mechanistic *in silico* model (Li et al., 2019b) based on a series of modification of O’Hara-Rudy (ORd) human ventricular myocyte model (O’hara et al., 2011).

However, a few groups have raised questions on the superiority in accuracies of model-driven *in silico* approaches. (Mistry et al., 2015; Mistry, 2017; Parikh et al., 2017; Mistry, 2018; Parikh et al., 2019; Mistry, 2019a) Especially, Mistry asserted that Bnet, a simple linear metric using the net difference between inward and outward ion channel blocking, has predictive power similar to that of TMS. (Mistry, 2019a) Mistry questioned the usefulness of the complicated *in silico* approaches proposed by CiPA if the performance to assess the proarrhythmic risk is similar, although the CiPA researchers asserted the superiority of *in silico* approaches that consider the trapping of the hERG and other channels through rigorous validation of the model (Li et al., 2019b).

In this report, we compared the performance of Bnet and TMS in discerning TdP risks of the whole 28 CiPA drugs (12 training and 16 validation) to gain insight into potentials and limitations of *in silico* approaches by CiPA.

## Methods

### Channel Block Data to Calculate Bnet

The CiPA have chosen 12 training and 16 validation drugs which have been classified by a team of clinical cardiologists and electrophysiologists into three categories according to the known TdP risks (high, intermediate, and low risk) (Colatsky et al., 2016).

To compare the relationship between TMS and Bnet of the 28 drugs, we first collected the ion channel block data (IC50 and Hill coefficients by the drugs) that were used to estimate the TMS (Li et al., 2019b). They were used to calculate Bnet values. Because the CiPA aimed to automate the assays by using high-throughput patch-clamp systems (HTS) (Sager et al., 2014), hybrid patch-clamp data collected using both manual (for hERG channel) and automated (other channels) methods were compared with data from the manual method for all channels. The performance of the hybrid and manual methods seemed equivalent (Li et al., 2019b). However, we picked the TMS values obtained from the manual method that has long been used as a standard in patch clamp studies.

In the many ion channels, only the four channels that were finally chosen by CiPA as significantly influencing the qNet and TMS: rapidly activating delayed rectifier potassium current (I_Kr_), late sodium current (INaL), L-type calcium current (ICaL), and peak sodium current (INa) (Li et al., 2019b). Thus, for the calculation of Bnet, we used the IC50 and Hill coefficients for the four channels identical to those used to estimate qNet and TMS by CiPA. Those for the 12 training drugs were retrieved from the report by Crumb et al. (2016) that was utilized by Li et al. (2019b) In the case of the 16 validation drugs, the CiPA researchers did not use published data but have performed patch-clamp studies on their own. (Li et al., 2019b) Thus, we retrieved the IC50 and Hill coefficients for INaL, ICaL, and INa channels from the report by Li et al. However, the CiPA researchers did not use simple channel block, but employed a channel-trapping model in the case of the hERG channel and the IC50 or Hill coefficients for hERG channel for the 16 validation drugs were not mentioned in their report at all. (Li et al., 2019b). Thus, we had to search other published data (**Table 1**) to replace those for I_Kr_ (hERG) of the 16 validation drugs.

**Table 1:** IC50 and Hill coefficient values of the hERG channel retrieved from the literature to calculate Bnet_5×Cma__x_ for the 16 validation drugs.

Compound	IC50 (µM)	Hill coefficient	Model	Literature	Temperature (°C)	Technique
Ibutilide	2	≒1	XO	(Lin et al., 2008)	21.5	Voltage-clamp 2-electrode
Azimilide	0.61	1	CHO	(Walker et al., 2000)	22	Whole-cell PC
Disopyramide	7.23	0.89	CHO	(Paul et al., 2001)	36	Whole-cell PC
Domperidone	0.057	0.99	HEK	(Claassen and Zünkler, 2005)	21	Whole-cell PC
Droperidol	0.0322	1.39	HEK	(Drolet et al., 1999)	22.5	Whole-cell PC
Pimozide	0.001	1.1	HEK	(Kirsch et al., 2004)	35	Whole-cell PC
Astemizole	0.0013	0.95	HEK	(Tarantino et al., 2005)	35	Whole-cell PC
Clozapine	2.5	0.82	HEK	(Lee et al., 2006)	35	Whole-cell PC
Clarithromycin	750	1.7	CHO	(Abbott et al., 1999)		Whole-cell PC
Risperidone	0.167	1	CHO	(Kongsamut et al., 2002)	23	Whole-cell PC
Metoprolol	145	1.1	HEK	(Kawakami et al., 2006)	23	Whole-cell PC
Tamoxifen	1.2	1.4	HEK	(Chae et al., 2015)	23	Whole-cell PC
Nifedipine	>50		HEK	(Zhang et al., 1999)	23	Whole-cell PC
Nitrendipine	10			(Redfern et al., 2003)		
Loratadine	173		HEK	(Crumb, 2000)	36 ± 1	Whole-cell PC
Vandetanib	1.15	0.76	HEK	(Lee et al., 2018)	37 ± 0.5	Whole-cell PC

CHO, Chinese hamster ovary cells; HEK, human embryonic kidney (HEK293) cells; Whole-cell PC, Whole-cell voltage-clamp recordings.

### Calculation of Percentage Block and Bnet_5×Cmax_

The percentage block (%block) against a repolarization or depolarization ion-channel inputted into the Bnet_5×Cmax_ model was calculated using the mean maximal concentration observed (C_max_) corrected for plasma protein binding (thus, unbound concentration)(Mistry, 2018).

$$\%\text{block}\;=\;\frac{100\;\times {\left(5\times C_{\max}\right)}^{\mathrm{Hill}}}{{\left(5\times C_{\max}\right)}^{\mathrm{Hill}}\;+\;\mathrm{IC}50^{\mathrm{Hill}}}$$

Bnet_5×Cmax_ was defined as the net difference in %block of the four most influential channels on the AP shape (%block of hERG channel − sum of %blocks of the other channels) at 5× C_max_ (**Supplementary Table 1**).

$$\mathrm{Bne}t_{5\times \;C_{\max}}\;\left(\%\right)=\sum_{i=0}^{n}R_{i}-\sum_{j=0}^{m}D_{j}$$

where Ri and Dj represent the %block against repolarization (I_Kr_) and depolarization (INaL, ICaL, and INa) ion-channels, respectively for a specific drug.

There are three major differences between the original Bnet (Mistry, 2018) and Bnet_5×Cmax_. First, compared to the original Bnet proposed by Mistry, %block in our study (Bnet_5×Cmax_) took Hill coefficient into consideration. Second, the original Bnet proposed by Mistry did not include INa, we included it because the four channels have been selected to calculate TMS by CiPA researchers. Third, the original Bnet used values at 1× C_max_ but Bnet_5×Cmax_ used values at 5× C_max_.

### Calculation of Dynamic Bnet

We compared TMS and Bnet_5×Cmax_ with “dynamic Bnet” (Mistry, 2019a), which reflects hERG dynamics as TMS used. We utilized the publicly available data set that Mistry provided (Mistry, 2019b) and in the data set, hERG dynamics was included into Bnet by replacing the static hERG block with the dynamic hERG blocking using IC50 and maximal inhibition at the 1× C_max_.

### Torsade Metric Score

The TMS, mean of qNet values at 1×, 2×, 3×, or 4× C_max_ derived from the CiPAORdv1.0 model was digitized from a report by Li et al. (2019b). As mentioned in the previous section, only the TMS values from manually measured data were collected for comparison.

### Ranking Performance Measures

Statistical analysis was performed using R Statistical Software version 3.6.0 (R Core Team, 2019). The ROC AUC (receiver operating characteristic curve area under the curve) (Zou et al., 2007) for TMS and Bnet_5×Cmax_ was calculated based on the known risk classifier. A logistic regression analysis using maximum likelihood estimation of the metric and the torsadogenic risk categories was performed by the rms R package (Harrell, 2019).

## Results

### Risk Misspecification by TMS and Bnet_5×Cmax_ in the Validation Drug Data Set

**Figure 1:**
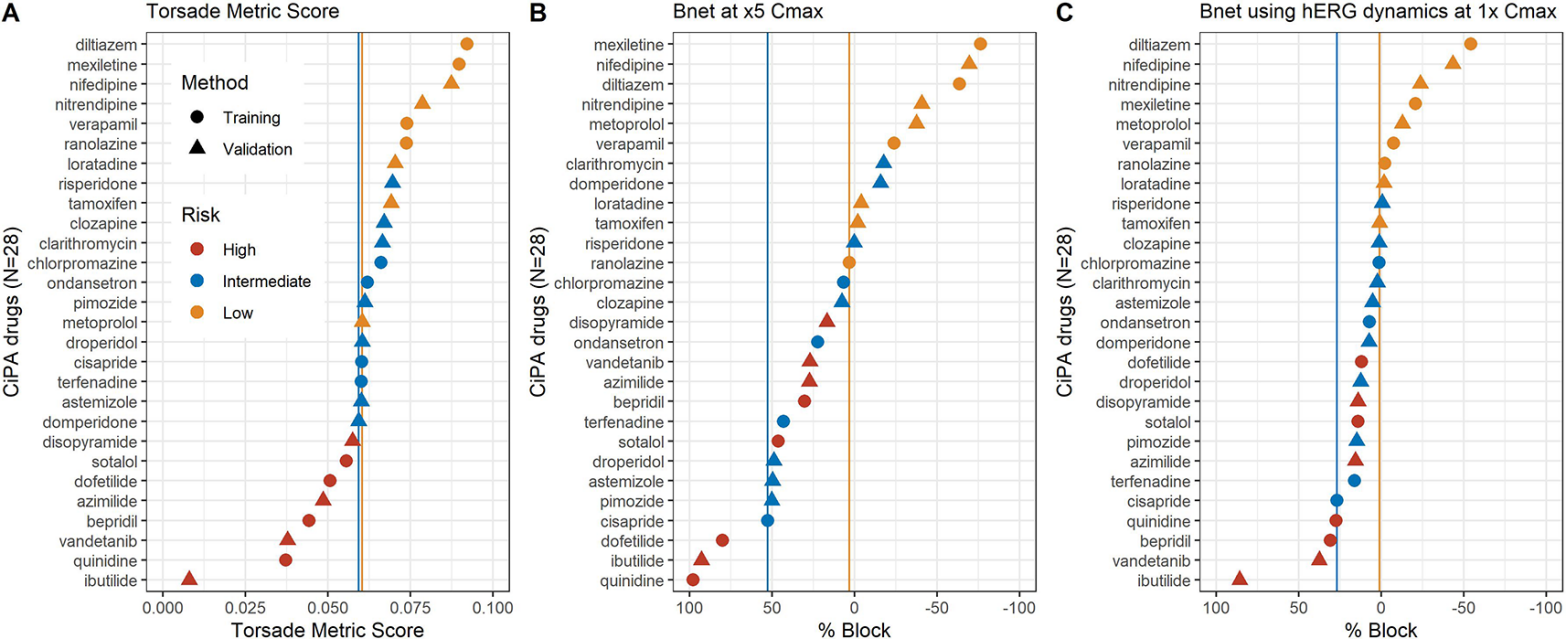
**(A)** median TMS (torsade metric score), **(B)** Bnet_5×Cmax_, and **(C)** Bnet using hERG dynamics at 1× Cmax for the 28 CiPA drugs by data sets (training or validation drugs) and risk categories (high, intermediate, or low risk). The 28 drugs are sorted according to the median TMS or Bnet values in each panel. The two vertical lines indicate borderlines dividing low- and intermediate-risk clusters (yellow) and intermediate and high-risk clusters (blue).

The TMS and Bnet_5×Cmax_ of all 12 training drugs tested with Crumb’s data (Crumb et al., 2016) were accordant to the risk categories (low vs. intermediate/high) (**Figure 1**). In the case of the 16 validation drugs, there were a few mismatches in categories both in the TMS (**Figure 1A**) and Bnet_5×Cmax_ methods (**Figure 1B**): tamoxifen and metoprolol (low-risk drugs) were located in the intermediate-risk cluster in the TMS and clarithromycin, domperidone, and risperidone (intermediate-risk drugs) were located in the low-risk cluster in the Bnet_5×Cmax_. Dynamic Bnet using hERG dynamics decreased the misspecification but a drug of the validation data set, risperidone, still was misclassified (**Figure 1C**).

### Strong Correlation Between TMS and Bnet_5×Cmax_ in the Training Drug Data Set

**Figure 2:**
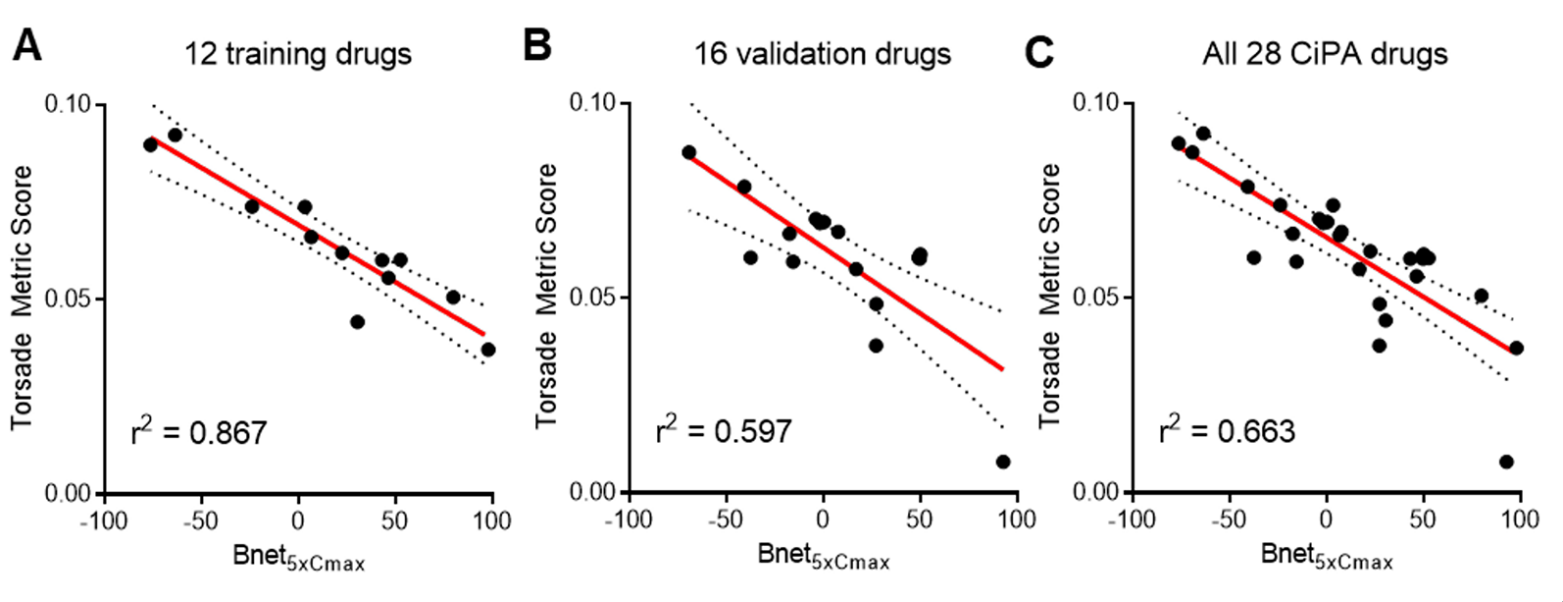
Correlation between Bnet_5×Cmax_ and median TMS (torsade metric score, the average of qNet at 1–4× C_max_) of **(A)** 12 training drugs, **(B)** 16 validation drugs, and **(C)** all 28 CiPA drugs. C_max_, peak plasma concentration.

Bnet_5×Cmax_ has shown performance similar to that of TMS as they are correlated with each other (*r* ^2^ = 0.663) (**Figure 2**). The correlation between Bnet_5×Cmax_ and TMS was stronger in the training drugs (*r* ^2^ = 0.867) than in the validation drugs (*r* ^2^ = 0.597), suggesting that the training drugs may possess better *in vitro* (patch-clamp study) data quality.

### Performance Comparison: TMS and Bnet_5×Cmax_

Both TMS and Bnet_5×Cmax_ seemed to discriminate low proarrhythmic risk drugs from intermediate/high-risk drugs quite well because the TMS and Bnet_5×Cmax_ values of low-risk drugs were significantly different (*t*-test, *p* < 0.005, and *p* < 0.001, respectively) from those of intermediate- and high-risk drugs (**Supplementary Figure 1**). The ranking performance measure for TMS and Bnet_5×Cmax_ was evaluated using ROC AUC (low vs. intermediate/high risk), and the values were 0.956 and 0.959, respectively (**Table 2**, **Supplementary Figure 2**).

**Table 2:** Prediction performance for 28 CiPA drugs of TMS, Bnet_5×Cma__x_, and Dynamic Bnet.

Performance metric	TMS	Bnet_5×Cmax_	Dynamic Bnet
ROC AUC (low vs. intermediate/high risk)	0.956	0.959	0.994
ROC AUC (low/intermediate vs. high risk)	0.990	0.844	0.925
*R* ^2^*	–	0.662	0.878
χ^2^ statistic^†^	41.73	23.70	33.55

*Coefficient of determination with torsade metric score and Bnet_5×Cmax_ or dynamic Bnet.

^†^Univariable logistic regression analysis to assess the correlation between the metric and the torsadogenic risk categories.

The ROC AUC (Low/Intermediate Vs. High Risk) and χ^2^ Statistic Derived From Univariable Logistic Regression Analysis of TMS Were Higher Than Those of Bnet_5×Cmax_ Suggesting That TMS Outperforms Bnet_5×Cmax_ in Discriminating Intermediate- and High-Risk Drugs (**Table 2**).

## Discussion

This is the first study to examine the performance of Bnet_5×Cmax_, a simple metric calculated as the gap in blocking four representative channels by 28 CiPA drugs. We showed that Bnet_5×Cmax_ provided predictability comparable to the large-scale mechanistic model.

The therapeutic C_max_ value directly affects the TMS and the Bnet metric. The TMS is calculated by averaging qNet values at 1×, 2×, 3×, or 4× C_max_ and Bnet_5×Cmax_ is calculated based on %block at 5× C_max_. We have first screened Bnet for 1×, 5×, and 10× C_max_ and the Bnet_5×Cmax_ showing the best performance was further used in our study (**Supplementary Table 2**).

Although the TMS and Bnet_5×Cmax_ of all 12 training drugs tested with Crumb’s data (Crumb et al., 2016) were in the exact order of risk categories pre-defined by previous reports (Shah, 2005), the data based on the 16 validation drugs showed a few incorrect predictions both in TMS and Bnet_5×Cmax_. This misspecification seems to have been caused by the patch-clamp experiments on the validation drugs not as qualified as in the 12 training drugs.

The reliability of patch-clamp experiment data is known to be highly variable by laboratories and skillfulness of the experimenter. Thus, measurement of IC50 and Hill coefficients using the patch-clamp method performed by well-trained personnel appears critical for the appropriate assessment of both TMS and Bnet, regardless of using the silico method. The CiPA’s attempt to estimate TMS with combined *in vitro* and *in silico* approaches is worthwhile in that the variability in multiple channel blocking is rigorously validated. Nonetheless, the performance of TMS is also dependent on the quality of patch-clamp experiment data for the ion channels that are input into the *in silico* simulation step.

The major limitation of Bnet is the inability to discriminate drugs with atypical binding kinetics (Li et al., 2019a). Because the hERG trapping observed in some drugs is not measured by the conventional ion channel blocking assay, CiPA has used the dynamic-hERG binding model for the data obtained using the Milnes protocol (Li et al., 2017). When the conventional IC50 is used to calculate Bnet for drugs that are significantly trapped in the hERG channel (e.g., dofetilide, bepridil, and terfenadine exemplified by Li et al. (2017)), their Bnet would be underestimated. However, the three drugs have shown Bnet values high enough to fall in the “intermediate/high” cluster in our study (**Figure 1**).

Recently, Mistry calculated “dynamic Bnet” (Mistry, 2019a) using hERG dynamic IC50 which may partly reflect binding kinetics and showed the higher correlation of dynamic Bnet with TMS of the 28 CiPA drugs (*r* ^2^ = 0.86) than the conventional Bnet_5×Cmax_ (*r* ^2^ = 0.66) presented in **Figure 2** in this report. The ROC AUC values of low- versus intermediate/high-risk for TMS, Bnet_5×Cmax_, and dynamic Bnet were 0.956, 0.959, and 0.994, respectively. Although dynamic Bnet showed the best performance, it also requires the additional *in silico* approach and the time and resources spent to acquire the metric in the discovery or preclinical stage may still be substantial. The Bnet_5×Cmax_ can be a straightforward, accessible, and simple screening tool to discern the low-risk drugs.

The highest prediction performance of low/intermediate-risk versus high-risk drugs was observed in the TMS (ROC AUC = 0.99, **Table 2**). However, in the actual early development process, drug candidates with an intermediate risk often cannot survive to the next development step, and we believe that this limitation of poor discerning between intermediate and high risks may not affect go/no-go decision at the early stage in almost of therapeutic areas except for antiarrhythmics.

The Bnet_5×Cmax_ metric may be used as a simple screening biomarker in drug discovery and early development. We demonstrated that the Bnet_5×Cmax_ (or Bnet at concentrations regarded high enough when no Cmax data are available) provides initial information whether a candidate is at low proarrhythmic risk or not. For a candidate worthy of further development even with intermediate/high risk according to the Bnet_5×Cmax_ metric, *in silico* approaches proposed by CiPA or dynamic Bnet may be helpful.

## Author’s Note

The abstract was submitted to Safety Pharmacology Society Anual Meeting 2019, held in Barcelona, Spain from September 23rd to 26th, 2019.

## Data Availability Statement

Publicly available datasets were analyzed in this study. This data can be found here: https://ascpt.onlinelibrary.wiley.com/doi/abs/10.1002/cpt.1184 and https://github.com/HiteshBMistry/Re-analysis-of-CiPA/blob/master/dynamic_qnet_versus_dynamic_bnet.csv.

## Author Contributions

SeH, K-SK, H-AL, and D-SY contributed to the acquisition of data and provided experimental data guidance. SuH, SeH, and D-SY contributed to designing the work and carried out simulations. SuH, SeH, and D-SY contributed to the analysis and interpretation of the data and writing of the manuscript.

## Funding

This research was supported by a grant (18182MFDS406) from Ministry of Food and Drug Safety in 2018.

## Conflict of Interest

The authors declare that the research was conducted in the absence of any commercial or financial relationships that could be construed as a potential conflict of interest.
