# Supplementary material for: Usefulness of Bnet, a Simple Linear Metric in Discerning Torsades De Pointes Risks in 28 CiPA Drugs
Source: Front Pharmacol. 2019 Nov 26;10:1419. doi: 10.3389/fphar.2019.01419 (PMC6889857; doi:10.3389/fphar.2019.01419)
Supplement: Supplementary file 1 [file DataSheet_1.docx]

# Supplementary Data

**Supplementary Table 1.** Bnet_5xCmax_, 5x C_max_, percentage block (%), IC50s (ng/mL) and Hill coefficients of the four channels for training drugs (N=12) and validation drugs (N=16)

|  | Parameter | INaL | ICaL | INa | IKr(hERG) | Bnet_5xCmax_  (%) | 5x C_max_ (uM) |
| --- | --- | --- | --- | --- | --- | --- | --- |
| **Training**  dofetilide | Block(%) | 0.0 | 0.0 | 0.4 | 79.9 | 79.5 | 0.01 |
|  | IC50 (Hill) | 126.0 (1.1) | 44.5 (3.6) | 1.4 (1.1) | 0.0 (0.6) |  |  |
| bepridil | Block(%) | 3.4 | 13.6 | 3.0 | 52.3 | 32.3 | 0.17 |
|  | IC50 (Hill) | 1.8 (1.4) | 2.8 (0.7) | 3.0 (1.2) | 0.1 (0.9) |  |  |
| sotalol | Block(%) | 0.0 | 1.8 | 0.2 | 46.4 | 44.3 | 73.45 |
|  | IC50 (Hill) | 3,280.0 (4.8) | 7,130.0 (0.9) | 112,000.0 (0.9) | 86.4 (0.9) |  |  |
| quinidine | Block(%) | 66.8 | 33.3 | 59.9 | 97.9 | -62.0 | 16.19 |
|  | IC50 (Hill) | 9.5 (1.3) | 53.5 (0.6) | 12.4 (1.5) | 0.3 (1.0) |  |  |
| cisapride | Block(%) | 0.0 | 0.0 | 0.0 | 52.6 | 52.6 | 0.01 |
|  | IC50 (Hill) | 9,260.0 (6.3) | 1,030.0 (4.8) | 1,790.0 (0.7) | 0.0 (1.3) |  |  |
| terfenadine | Block(%) | 1.3 | 8.7 | 0.0 | 50.8 | 40.8 | 0.02 |
|  | IC50 (Hill) | 14.9 (0.7) | 0.7 (0.7) | 1.7 (2.4) | 0.0 (0.6) |  |  |
| ondansetron | Block(%) | 3.5 | 6.6 | 0.2 | 31.8 | 21.5 | 0.70 |
|  | IC50 (Hill) | 19.3 (1.0) | 22.7 (0.8) | 38.5 (1.6) | 1.5 (1.0) |  |  |
| chlorpromazine | Block(%) | 4.8 | 3.9 | 0.1 | 16.9 | 8.1 | 0.19 |
|  | IC50 (Hill) | 4.6 (0.9) | 8.3 (0.9) | 4.6 (2.1) | 1.1 (0.9) |  |  |
| verapamil | Block(%) | 0.0 | 68.0 | 0.0 | 44.3 | -23.8 | 0.41 |
|  | IC50 (Hill) | 24.1 (2.0) | 0.2 (1.1) | 2,590.0 (3.5) | 0.5 (1.1) |  |  |
| ranolazine | Block(%) | 54.8 | 0.0 | 3.8 | 58.1 | -0.6 | 9.74 |
|  | IC50 (Hill) | 7.9 (1.0) | 900.0 (3.9) | 53.3 (1.9) | 6.5 (0.8) |  |  |
| mexiletine | Block(%) | 76.1 | 34.7 | 29.1 | 0.0 | -139.9 | 20.65 |
|  | IC50 (Hill) | 9.0 (1.4) | 38.9 (1.0) | 26.1 (3.8) | - |  |  |
| diltiazem | Block(%) | 8.1 | 77.1 | 0.3 | 13.0 | -72.5 | 0.61 |
|  | IC50 (Hill) | 21.6 (0.7) | 0.1 (0.7) | 36.9 (1.4) | 6.6 (0.8) |  |  |
| **Validation**  vandetanib | Block(%) | 0.1 | 24.6 | 0.0 | 52.0 | 27.2 | 1.28 |
|  | IC50 (Hill) | 3,790.0 (0.8) | 6.1 (0.7) | 80.9 (1.9) | 1.2 (0.8) |  |  |
| ibutilide | Block(%) | 0.0 | 3.2 | 0.0 | 96.2 | 92.9 | 0.70 |
|  | IC50 (Hill) | 287.0 (2.4) | 37.0 (0.9) | 24.1 (2.3) | 0.0 (1.0) |  |  |
| azimilide | Block(%) | 1.4 | 7.1 | 0.7 | 36.5 | 27.3 | 0.35 |
|  | IC50 (Hill) | 2,940.0 (0.5) | 13.2 (0.7) | 363.0 (0.7) | 0.6 (1.0) |  |  |
| disopyramide | Block(%) | 0.0 | 18.2 | 0.6 | 35.6 | 16.8 | 3.71 |
|  | IC50 (Hill) | 377.0 (2.1) | 32.9 (0.7) | 192.0 (1.3) | 7.2 (0.9) |  |  |
| domperidone | Block(%) | 0.0 | 53.1 | 0.0 | 37.5 | -15.6 | 0.10 |
|  | IC50 (Hill) | 225.0 (2.1) | 0.1 (0.5) | 41.9 (1.5) | 0.2 (1.0) |  |  |
| droperidol | Block(%) | 0.0 | 0.4 | 0.0 | 49.4 | 49.0 | 0.03 |
|  | IC50 (Hill) | 33.8 (2.8) | 3.2 (1.2) | 36.6 (2.5) | 0.0 (1.4) |  |  |
| pimozide | Block(%) | 0.0 | 17.8 | 2.0 | 69.9 | 50.1 | 0.002 |
|  | IC50 (Hill) | 1.9 (1.9) | 0.1 (0.5) | 10.2 (0.5) | 0.0 (1.1) |  |  |
| clozapine | Block(%) | 0.0 | 7.1 | 2.0 | 16.8 | 7.7 | 0.36 |
|  | IC50 (Hill) | 73.6 (2.0) | 5.5 (0.9) | 257.0 (0.6) | 2.5 (0.8) |  |  |
| risperidone | Block(%) | 0.0 | 4.7 | 0.0 | 5.1 | 0.4 | 0.01 |
|  | IC50 (Hill) | 11,400.0 (5.8) | 1.5 (0.6) | 534.0 (0.7) | 0.2 (1.0) |  |  |
| astemizole | Block(%) | 0.0 | 0.1 | 0.2 | 50.0 | 49.8 | 0.001 |
|  | IC50 (Hill) | 10.3 (2.3) | 0.6 (1.2) | 5.4 (0.8) | 0.0 (1.0) |  |  |
| clarithromycin | Block(%) | 0.0 | 16.5 | 1.0 | 0.0 | -17.4 | 6.03 |
|  | IC50 (Hill) | 1,810.0 (3.0) | 38.1 (0.9) | 1,090.0 (0.9) | 750.0 (1.7) |  |  |
| tamoxifen | Block(%) | 0.0 | 4.6 | 0.4 | 3.2 | -1.8 | 0.11 |
|  | IC50 (Hill) | 3,640.0 (4.0) | 5.7 (0.8) | 84.0 (0.8) | 1.2 (1.4) |  |  |
| metoprolol | Block(%) | 5.7 | 4.0 | 32.3 | 4.5 | -37.5 | 9.00 |
|  | IC50 (Hill) | 630.0 (0.7) | 3,280.0 (0.5) | 30.3 (0.6) | 145.0 (1.1) |  |  |
| loratadine | Block(%) | 0.0 | 3.9 | 0.0 | 0.0 | -3.9 | 0.002 |
|  | IC50 (Hill) | 192.0 (2.1) | 0.7 (0.6) | 113.0 (1.4) | 173.0 (1.0) |  |  |
| nitrendipine | Block(%) | 0.0 | 39.4 | 1.4 | 0.2 | -40.7 | 0.02 |
|  | IC50 (Hill) | 70.7 (3.2) | 0.0 (0.5) | 22.4 (0.6) | 10.0 (1.0) |  |  |
| nifedipine | Block(%) | 0.0 | 69.3 | 0.1 | 0.1 | -69.3 | 0.04 |
|  | IC50 (Hill) | 45.6 (4.5) | 0.0 (0.7) | 27.6 (1.1) | 50.0 (1.0) |  |  |

Abbreviation: C_max_, peak plasma concentration; IC50, half maximal inhibitory concentration; IKr, rapidly activating delayed rectifier potassium current; INaL, late sodium current; ICaL, L-type calcium current; INa, peak sodium current.

**Supplementary Table 2.** Coefficient of determination with torsade metric score, receiver operating characteristic, and logistic regression analysis of Bnet at the 1x C_max_, 5x C_max_, 10x C_max_ using all drugs (N=28)

|  | Bnet_1xCmax_ | Bnet_5xCmax_ | Bnet_10xCmax_ |
| --- | --- | --- | --- |
| ROC AUC^*^ | 0.918 | 0.959 | 0.923 |
| R^2^ | 0.733 | 0.662 | 0.415 |
| χ^2^ statistic^†^ | 22.59 | 23.70 | 13.98 |

^*^ receiver operating characteristic area under the curve of low vs. intermediate/high risk

^†^ univariable logistic regression analysis to assess the correlation between Bnet and the torsadogenic risk categories


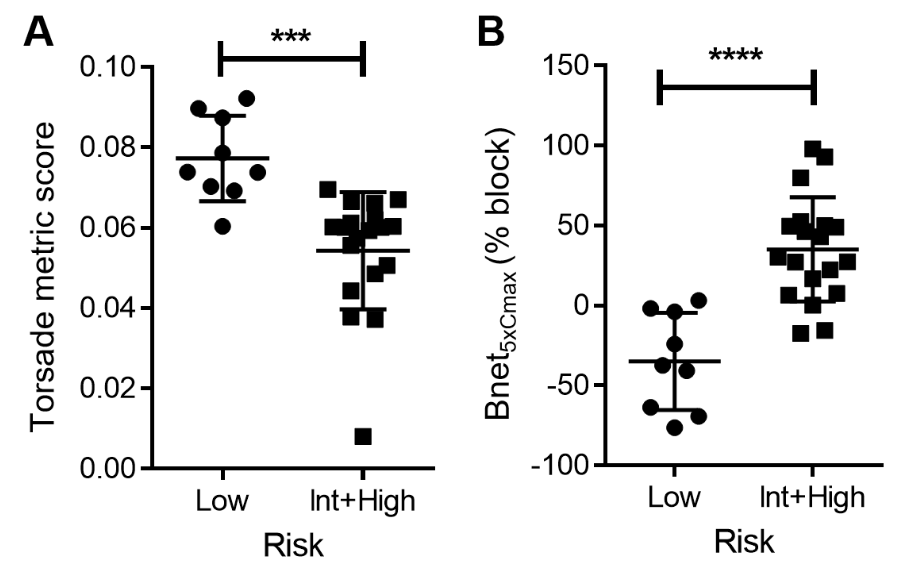


**Supplementary Figure 1.** Scatter plot of A) median TMS and B) Bnet_5xCmax_ by risk categories (low-risk vs. intermediate- and high-risk). Cmax, peak plasma concentration. P-values are calculated using a two-tailed t-test. (*** denotes a p-value of <0.005; and **** denotes a p-value of <0.001.)


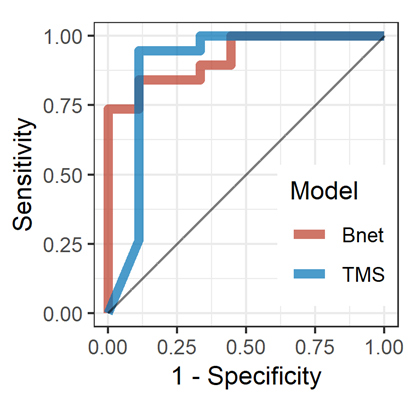


**Supplementary Figure 2.** Receiver operating characteristic curves for TMS and Bnet_5xCmax_.
